# LncRNA NEAT1 Interacted With DNMT1 to Regulate Malignant Phenotype of Cancer Cell and Cytotoxic T Cell Infiltration via Epigenetic Inhibition of p53, cGAS, and STING in Lung Cancer

**DOI:** 10.3389/fgene.2020.00250

**Published:** 2020-03-31

**Authors:** Fang Ma, Yi-Yu Lei, Meng-Ge Ding, Li-Hua Luo, Yang-Chun Xie, Xian-Ling Liu

**Affiliations:** ^1^Department of Oncology, The Second Xiangya Hospital, Central South University, Changsha, China; ^2^Cancer Center, Union Hospital, Tongji Medical College, Huazhong University of Science and Technology, Wuhan, China

**Keywords:** NEAT1, DNMT1, cGAS/STING pathway, lung cancer, mechanisms

## Abstract

**Purpose:**

Lung cancer is the main cause of cancer-related mortality worldwide. We report here the biological role of nuclear paraspeckle assembly transcript 1 (NEAT1) in the pathogenesis of lung cancer and the underlying mechanisms.

**Methods:**

Reverse transcription–quantitative polymerase chain reaction (RT-qPCR) and Western blotting analysis were used to evaluate expression of mRNA and protein. RNA immunoprecipitation (RIP) assay, chromatin immunoprecipitation followed by qPCR analysis, and reporter assay were used to detect DNA/RNA and protein binding. Tumor-infiltrating lymphocytes were assessed with hematoxylin-eosin staining. Cytotoxic T cell infiltration was evaluated with flow cytometric analysis and immunohistochemistry (IHC) staining. The changes of cell viability and cell invasive and migratory ability were analyzed by MTT (3-(4,5-dimethylthiazol-2-yl)-2,5-diphenyltetrazolium bromide), colony formation, and Transwell assays, respectively. Syngeneic tumor model was set up to evaluate antitumor effect.

**Results:**

The results showed that NEAT1 was overexpressed in lung cancer tissues and cancer cell lines. This aberrant expression was closely related with tumor stage and lymph node metastasis. Tumor sample with high CD8^+^ showed lower NEAT1 expression. *In vitro* studies displayed that inhibition of NEAT1 with shRNA resulted in suppression of survival and migration/invasion of lung cancer cells. On the other side, NEAT1 was found to promote tumor growth via inhibiting cytotoxic T cell immunity in syngeneic models. Finally, NEAT1 was found to interact with DNMT1, which in turn inhibited P53 and cyclic GMP-AMP synthase stimulator of interferon genes (cGAS/STING) expression.

**Conclusion:**

Our findings demonstrated that NEAT1 interacted with DNMT1 to regulate cytotoxic T cell infiltration in lung cancer via inhibition of cGAS/STING pathway. The results provided the novel mechanistic insight into the pathogenesis of lung cancer.

## Introduction

Lung cancer is the main cause of cancer-related mortality worldwide. Surgery and chemotherapy are the major treatments for lung cancer. However, the 5-year overall survival of non-small cell lung cancer (NSCLC) patients is very low (Brody, 2014). Non-small cell lung cancer is the most common subtype of lung cancer that includes large cell carcinoma, adenocarcinoma, and squamous cell carcinoma. Small cell lung cancers account for 10–15% of lung cancers and tend to spread quickly (de Sousa and Carvalho, 2018).

Lung cancer tumorigenesis and development rely on the evolving genomics and molecular properties of cancer cells as well as the interplay with the immune system (Rafei et al., 2017). The immune system can recognize tumor-associated antigens and regulate the body’s ability to attack tumor cells. To escape immunosurveillance, nascent malignant cells may develop diverse mechanisms, including reducing antigenicity so that antitumor lymphocytes fail to detect transformed cells, eliminating immunogenicity by upregulating immunoinhibitory molecules, and recruiting immunosuppressive cells to establish an immunosuppressive microenvironment (Liu and Cao, 2016). Therapeutic progress for NSCLC can largely be attributed to the tumor immune biology and the development of newer generations of cancer immunotherapies (Rafei et al., 2017). Along with the rapid development of immunotherapy, traditional chemotherapy in wild-type (pan-negative) advanced NSCLC has been challenged by single-agent checkpoint inhibitors from second-line treatment to first-line treatment. Monoclonal antibodies targeting programmed cell death ligand 1, cytotoxic T-lymphocyte–associated antigen 4, and programmed cell death 1 immune checkpoints have received regulatory approval in NSCLC (Dholaria et al., 2016).

The cyclic GMP-AMP synthase (cGAS) stimulator of interferon genes (STING) pathway induces the expression of type I interferons (IFNs) and other inflammatory cytokines to induce innate immunity for antimicrobial effects in response to viral and bacterial DNA (Pepin and Gantier, 2017). Besides pathogen recognition, cGAS-STING signaling is important for antitumor immunity because of the induction of type I IFN and activation of both innate and adaptive immunity (Corrales and Gajewski, 2015). The expression levels of these genes are negatively associated with their methylation levels in most of the detected cancer types (An et al., 2019).

DNMT1, along with DNMT2, DNMT3A, DNMT3B, and DNMT3L, belongs to DNA methyl transferase family, which regulate target gene expression by the CpG island methylation (Wu et al., 2019). Aberrant methylation, particularly in the promoter regions of tumor suppressor genes, inhibits gene expression and can facilitate human tumorigenesis (Zhou et al., 2014). It has been reported that cGAS and STING expression is suppressed by methylation in a pan-tumor analysis, indicating the possibility that cGAS and STING might be inhibited by DNMT1 (Konno et al., 2018).

Nuclear paraspeckle assembly transcript 1 (NEAT1) plays critical roles in the pathogenesis of various human malignancies (Yu et al., 2017). It was found to be overexpressed in human cancers (Li et al., 2018a). Its aberrant expression is closely interrelated with poorer clinicopathological parameters of lung cancer (Li et al., 2018b). Although it was reported that NEAT1 promotes the apoptosis, proliferation, and metastasis of lung cancer cells (Qi et al., 2018), the involvement of NEAT1 in immune oncology remains to be explored. Furthermore, NEAT1 epigenetically suppresses E-cadherin expression through association with G9a-DNMT1-Snail complex (Li and Cheng, 2018), which suggested that NEAT1 might exert protumor effect via DNMT1.

In the present study, the biological function and mechanism of NEAT1 in lung cancer were investigated. It was shown that NEAT1 was highly expressed in lung cancer tissue and cells. Nuclear paraspeckle assembly transcript 1 was involved in the lung cancer progression and cytotoxic T cell infiltration via binding DNMT1 to inhibit P53 and cGAS/STING pathways.

## Materials and Methods

### Clinical Sample Collection

A total of 32 lung cancer tissues and paired normal tissue specimens were collected from patients at the second Xiangya Hospital, Central South University, and frozen in liquid nitrogen. All the demographic and clinical characteristics are depicted in Table 1. All patients signed informed consent. Ethical approval was obtained from Ethical Committee of Central South University.

**TABLE 1:** Clinical pathological characteristics of 32 lung cancer patients.

Parameter	Cases (32)
**Gender**	
Male	14
Female	18
**TNM stage**	
I + II	20
III + IV	12
**Differentiation**	
Well	11
Moderate-Poor	21
**Lymph node metastasis**	
Yes	17
No	15
**Smoking**	
Yes	22
No	10
**Histological subtype**	
LUAD	12
LUSC	17
Big cell cancer	3

### Cell Culture

Human lung cancer cell lines A549, HCC1299, HCC827, NCI-H460, SK-MES-1, and NCI-H358; human normal bronchial epithelial cells 16HBE; and mouse lung cancer cell line M109 were purchased from the American Type Culture Collection (ATCC, Manassas, VA, United States). The cells were maintained in Dulbecco modified eagle medium (DMEM) supplemented with 10% fetal bovine serum (FBS) (Invitrogen, Carlsbad, CA), 100 mg/mL penicillin G (Invitrogen), and 100 U/mL streptomycin (Invitrogen) in an incubator (37°C, 5% CO_2_).

### Subcutaneous Syngeneic Lung Cancer Models

Male BALB/c mice were used at 6–8 weeks of age. All mice were obtained from Hunan SJA Laboratory Animal Co., Ltd. (Hunan, China). Mouse M109 syngeneic lung cancer cells in 100 μL phosphate-buffered saline (PBS) were implanted subcutaneously in the right flank of BALB/c mice. When the tumors were palpable, mice were randomized into two groups of five animals. Tumor volume were determined every 3 days in two dimensions using a caliper, and the volume was expressed in mm^3^ using the formula: *V* = 0.5 (*a* × *b*^2^), where *a* and *b* are the long and short diameters of the tumor, respectively. At the 21st day, mice were sacrificed, and the tumor tissues were obtained for further detections including Western blot analysis, flow cytometry, and reverse transcription-quantitative polymerase chain reaction (RT-qPCR). Animal studies were reviewed and approved by the Institutional Animal Care and Use Committee of Central South University.

### Flow Cytometry

Tumors were excised, weighed, and mechanically minced. Minced tumors were placed in gentle MACS Dissociator with Tumor Dissociation Kit for mouse tissues (Miltenyi Biotec, San Diego, CA, United States) to isolate immune and tumor cell subsets in accordance with the manufacturer’s directions. The cell suspension was passed through a 40-μm cell strainer (Falcon 352340) and washed twice. The responded cells were lysed with red blood cell lysis buffer (ACK) and incubated with mouse immunoglobulin G in FACS buffer for 15 min at 4°C. Tumor-infiltrating cells were stained with fluorochrome-conjugated anti–mouse antibodies, as well as appropriate isotype control antibodies. The following monoclonal antibodies and reagents were obtained from BD Bioscience (San Jose, CA, United States): anti-CD45 (30-F11, 1:200 dilution), anti-CD3 (clone 145-2c11, 1:200 dilution), anti-CD4 (GK1.5, 1:200 dilution), and anti-CD8 (clone 53–6.7, 1:200 dilution). Flow cytometry was carried out with LSRII flow cytometer (BD Biosciences, San Jose, CA, United States), and data were analyzed with FlowJo software (v.10.4; Tree Star, San Carlos, CA, United States).

### Western Blot

Total protein was isolated from cells or tumor tissues with ice-cold lysis buffer consisting of 50 mM Tris–HCl, pH 7.4, 120 mM NaCl, 5 mM EDTA, 0.5% Nonidet P-40, 0.1% Triton, and phosphatase and protease inhibitors. Protein concentration for each cell lysate was determined using a BCA protein assay kit (Pierce, Appleton, WI, United States), followed by loading equal amounts of protein into the wells of the sodium dodecyl sulfate-polyacrylamide gel electrophoresis (SDS-PAGE) gel. The proteins were subjected to SDS-PAGE and were transferred to polyvinylidene difluoride membrane, which was blocked overnight at 4°C using blocking buffer and incubated with dilutions of primary antibody against cGAS (D1D3G), p-STING (D8K6H), STING (D2P2F), p-TBK1 (D52C2), TBK1 (D1B4), p-IRF3 (E7J8G), and IRF3 (D6I4C) and secondary antibody against. All the antibodies were purchased from Cell Signaling Technology (Danvers, MA, United States). Bands were developed using Rapid Step^TM^ ECL Reagent (EMD Millipore, Billerica, MA, United States) according to the manufacturer’s instructions.

### Reverse Transcription-Quantitative Polymerase Chain Reaction

Total mRNA was isolated from cells and tissues with TRIzol Reagent method (Invitrogen, Cergy-Pontoise, France). Tumor samples and adjacent non-cancerous tissues were ground in liquid nitrogen before the addition of Trizol. Reverse transcription was carried out with RNA served as a template to synthesize cDNA. SYBR^®^ Green Real-Time PCR Master Mix (Thermo Fisher Scientific, Inc., Waltham, MA, United States) was utilized for PCR reaction. The primers used in PCR reactions are shown in Table 2.

**TABLE 2:** Primers list.

**Gene**	**Forward primer**	**Reverse primer**
Mouse NEAT1	GGGGCCACATTAATCACAAC	CAGGGTGTCCTCCACCTTTA
Human NEAT1	GTGGCTGTTGGAGTCGGTAT	TAACAAACCACGGTCCATGA
Mouse CXCL10	AGGACGGTCCGCTGCAA	CATTCTCACTGGCCCGTCAT
Human CXCL10	TTCTTAGTGGATGTTCTGACC	GTGTTTGGAATTGTATGTAGGT
Mouse CCL5	GAATACATCAACTATTTGG AGAT	TAGAGCAAGCAATGACAG
Human CCL5	CTGTATGACTCCCGGCTGAA	CCCAAGCTAGGACAAGAGCA
Mouse IFNβ	TCCAAGAAAGGACGAACATTCG	TGAGGACATCTCCCACGTCAA
Human IFNβ	GCCTCAAGGACAGGATGAAC	AGTCTCATTCCAGCCAGTGC
Human cGAS	GAAGAAACATGGCGGCTATC	TGAGGGTTCTGGGTACATACG
Human STING	CAGGCACTGAACATCCTCCT	ATATACAGCCGCTGGCTCAC
Human P53	GTGGAAGGAAATTTGCGTGT	AGCTGTTCCGTCCCAGTAGA
Mouse GAPDH	AGCCCAAGATGCCCTTCAGT	CCGTGTTCCTACCCCCAATG
Human GAPDH	CCAGGTGGTCTCCTCTGA	GCTGTAGCCAAATCGTTGT

### Cell Transfection

For construction of cell line overexpressing NEAT1, NEAT1 cDNA was amplified via PCR and inserted into pEGFPC3 (Clontech, Palo Alto, CA, United States) vector to establish the NEAT1 overexpression vector. Lipofectamine^®^ 3000 (Thermo Fisher Scientific, Inc.) was used to transfect cells according to the manufacturer’s protocol. The suppression of NEAT1 was performed by short-hairpin RNA (shRNA) interference purchased from GenePharma (Shanghai, China). Short-hairpin RNA and scrambled control RNA were transiently transfected into cancer cells line for 48 h.

### Cell Viability Assay

Cell viability was measured by 3-(4,5-dimethylthiazol-2-yl)-2,5-diphenyltetrazolium bromide) (MTT; Sigma, St. Louis, MO, United States) assay and colony formation assays. The cells were plated in 96-well plates at cell density of 5 × 10^3^ cells per well followed by treatment with MTT of 20 μL for 4 h. The supernatant was removed, and 150 μL dimethyl sulfoxide was added (Sigma). The absorbance of each sample was measured at 570 nm. The average of three repeated experiments was calculated. For colony formation assays, 500 cells were seeded into six-well plates and cultured for 10–14 days. The cells were washed with PBS, and visible colonies were fixed and stained with 0.5% crystal violet and methanol. The average of three repeated experiments was calculated.

### Transwell Assay

Cell invasion and migration were assessed with 24-well Transwell chambers coated with or without Matrigel (BD Biosciences) on the upper surface of the membrane with a pore size of 8 μm (Sigma). Briefly, cells (3 × 10^4^ cells/well) were seeded into the upper Transwell chamber. The lower chamber was soaked with 500 μL DMEM containing 10% FBS. After incubation for 24 h, the chambers were washed three times and fixed with 4% paraformaldehyde for 10 min. Finally, the chambers were stained with crystal violet, and the cells that passed through the membrane were counted visually under a microscope (Olympus, Tokyo, Japan).

### RNA Immunoprecipitation Assay

RNA immunoprecipitation (RIP) assay was performed according to manufacturer’s protocol (Millipore Corporation). Briefly, cells were harvested and lysed followed by incubation with the detection antibody (#5032; Cell Signaling Technology) at 4°C overnight with gentle rotation. The protein A/G beads (40 μL) were added and incubated with lysate for 1 h at 4°C to capture the complex. After washing in the buffer, RNA was isolated for detection of its concentration by RT-qPCR.

### Chromatin Immunoprecipitation Analysis

Chromatin immunoprecipitation (ChIP) analysis was carried out with ChromaFlash High-Sensitivity ChIP Kit (EpiGentek, #P-2027-24) following the manufacturer’s instructions. In brief, intact cells were treated with 1.0% formaldehyde to covalently link DNA and protein. After crosslinking, the cells were lysed in 0.1% SDS containing buffer. Genomic DNA fragmentation was obtained with Sonicate 9× for 10–20 s at 80% setting (Vibra-Cell Sonicator, Jiangsu, China). DNA-protein samples were incubated the DNMT1 antibody overnight at 4°C by constant rotation in the cold room followed by several rounds of washing with wash buffers. The digestions of samples were performed with RNAse and proteinase K. DNA was then purified using the column provided in the kit. DNA samples were analyzed by qPCR.

### Luciferase Reporter Assay

The report plasmid construction was performed by PCR amplification fragments of promoter region of TP53, CGAS, and STING1 fragments inserted into *Xho*I and *Kpn*I sites upstream of the firefly luciferase in the pGL3-promoter vector (Promega, Madison, WI, United States). Dual-Luciferase Reporter Assay (Promega) was performed to measure the firefly and Renilla luciferase activities.

### Immunohistochemistry Staining

The tumor tissues were fixed in 10% paraformaldehyde, decalcified in formic acid, and embedded in paraffin. Consecutive tumor sections (4 μm in thickness) were prepared and subjected to IHC staining. After deparaffinization and antigen retrieval, sections were incubated with antibodies against CD8 (144B, CST, 1:200) overnight followed by incubation with an appropriate secondary antibody for 1 h.

### Statistical Analysis

All data were presented as the mean of at least triplicate samples ± standard deviation. The data were analyzed with GraphPad Prism (GraphPad Software, San Diego, CA, United States). Statistical significance was evaluated with the one-way analysis of variance or Student *t*-test. *p* < 0.05 was considered statistically significant.

## Results

### Upregulation of NEAT1 in Lung Cancer Tissues and Cell Lines

**FIGURE 1:**
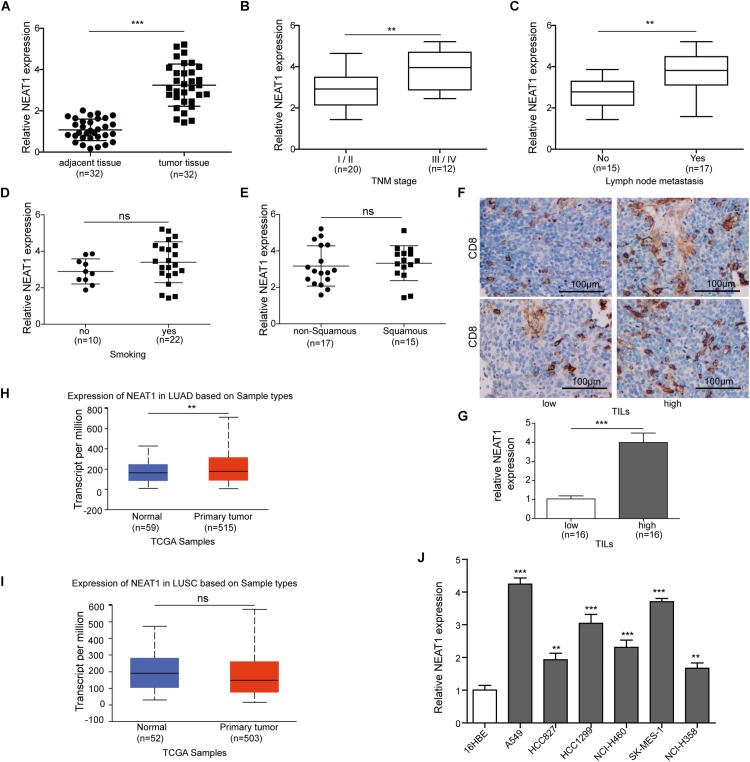
Nuclear paraspeckle assembly transcript 1 expression in lung cancer tissues and cell lines. **(A)** The NEAT1 mRNA expression level was detected in lung cancer tissues and adjacent normal tissues from 32 pairs of lung cancer patients using RT-qPCR. **(B)** The NEAT1 mRNA expression level was detected in lung cancer tissues with different tumor stage using RT-qPCR. **(C)** The NEAT1 mRNA expression level was detected in lung cancer tissues with different lymph node metastasis by using RT-qPCR. **(D)** The NEAT1 mRNA expression level was detected in smoker versus non-smoker groups using RT-qPCR. **(E)** The NEAT1 mRNA expression level was detected in non-squamous versus squamous cancer patients using RT-qPCR. **(F)** The representative image of CD8 staining in lung tumor sample. **(G)** The NEAT1 mRNA expression level is detected in samples with low tumor-infiltrating lymphocytes (TILs) versus those with high TILs using RT-qPCR. The NEAT1 expression in the samples “high TILs” has been normalized versus the samples “low TILs.” Panels **(H,I)** the NEAT1 mRNA expression level in LUAD and LUSC subtypes based on TCGA data set. **(J)** The expression levels of NEAT1 mRNA were measured by RT-qPCR analysis in different cell lines. **p < 0.01, ***p < 0.001. Data are expressed as the mean ± SD.

Reverse transcription qPCR analysis revealed that the expression of NEAT1 was remarkably increased in 32 lung cancer tissues compared with 32 respective adjacent non-cancerous tissues (Figure 1A). To further explore the significance of NEAT1 expression in lung cancer, the correlations between NEAT1 expression and clinicopathological features of lung cancer patients were determined. Higher expression of NEAT1 was associated with a TNM stage (Figure 1B) and lymph node metastasis (Figure 1C). The mRNA level of NEAT1 in the smoker group showed no significant difference relative to the non-smoker group (Figure 1D). Additionally, there was also no obvious difference in NEAT1 expression level between squamous versus non-squamous cancer patients (Figure 1E). The patients were also subdivided into two groups based on intratumor CD8^+^ T cell expression by IHC staining (Figure 1F). Patients with high expression of CD8 showed higher expression level of NEAT1 (Figure 1G). To validate our clinical analysis, we used The Cancer Genome Atlas (TCGA) database to confirm whether NEAT1 is correlated with NSCLC, stage, lymph node metastasis, and smoking. TCGA Lung Adenocarcinoma (LUAD) data showed that expression level of NEAT1 was upregulated in tumor tissues, which is in line with our 32 patients, whereas the expression has no significant difference between tumor and normal tissues based on lung squamous cell carcinoma (LUSC) data set (Figures 1H,I). This indicated us our data set might be small to draw conclusions on the NEAT1 expression levels between squamous and non-squamous cancer patients in Figure 1E. Both TCGA LUAD and TCGA LUSC also showed that there is no statistical difference of NEAT1 expression between the non-smoker and smoker groups (Supplementary Figures S3C,F), different stages (Supplementary Figures S3A,D), and nodal metastasis status (Supplementary Figures S3B,E). Consistently, the expression level of NEAT1 was upregulated in lung cancer cell lines when compared with that in human normal bronchial epithelial cells 16HBE (Figure 1J). These results suggest that NEAT1 is upregulated in lung cancer tissues and cell lines.

### Knockdown of NEAT1 Inhibits Cell Viability and Migration/Invasion of Lung Cancer Cells

**FIGURE 2:**
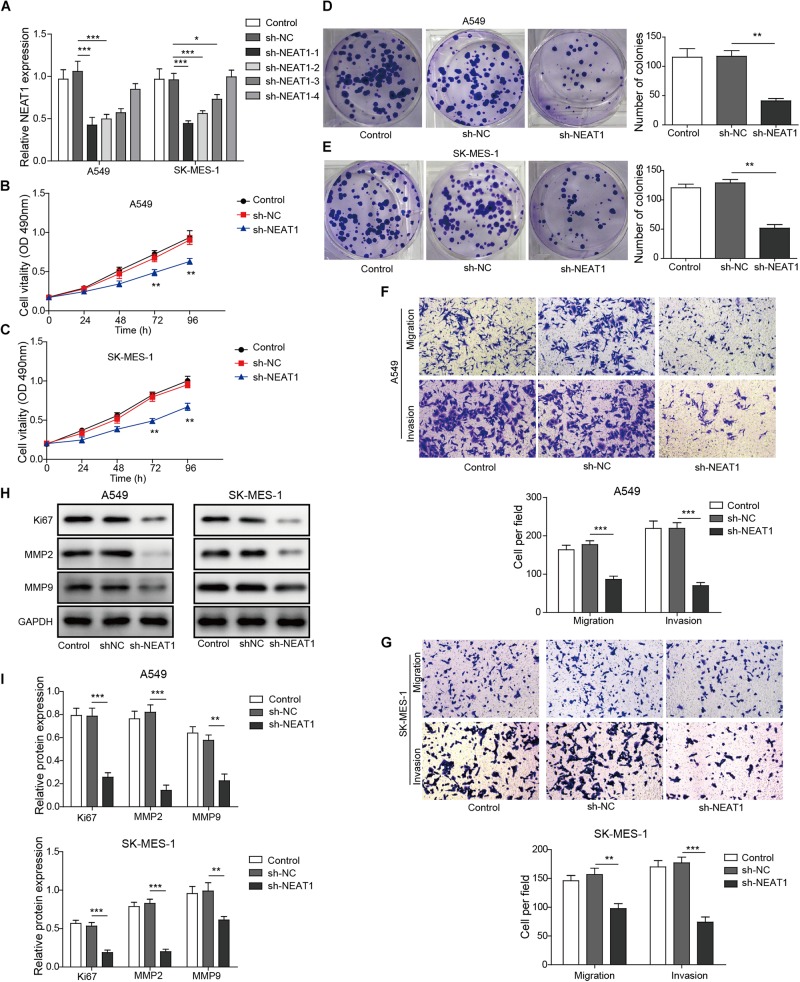
Down-regulation of NEAT1 suppresses the proliferation, migration, and invasion of lung cancer cells. **(A)** mRNA expression of NEAT1 was detected in A549 cells and SK-MES-1 cells when transfected with several specific shRNA of NEAT1 by using RT-qPCR. Panels **(B,C)** the proliferation of A549 cells and SK-MES-1 cells was analyzed after transfection with the specific shRNA for NEAT1 for 24, 48, 72, and 96 h by using MTT, respectively. Panels **(D,E)** the colony formation ability of A549 cells and SK-MES-1 cells was analyzed after transfection with the specific shRNA for NEAT1, respectively. Panels **(F,G)** the cell migration and invasion of A549 cells and SK-MES-1 cells were analyzed after transfection with the specific shRNA for NEAT1, respectively. Panels **(H,I)** the expression of Ki-67, MMP2, and MMP9 was measured after transfection with the specific shRNA for NEAT1 for 72 h by Western blot in A549 cells and SK-MES-1 cells, respectively. *p < 0.05, **p < 0.01, ***p < 0.001. Data are expressed as the mean ± SD.

The effect of NEAT1 on cell survival and migration/invasion was characterized in lung cancer cell lines with specific shRNA targeting NEAT1. The mRNA expression of NEAT1 was confirmed by qPCR, and shRNA-1 was used for further studies (Figure 2A). As observed in Figures 2B,C, knockdown of NEAT1 significantly suppressed the cell viability in A549 cells and SK-MES-1 cells in a time-dependent manner. Similarly, colony formation assays also displayed that inhibition of NEAT1 dramatically suppressed the ability of colony formation in A549 cells and SK-MES-1 cells (Figures 2D,E). The effect of NEAT1 on cell migration and invasion was measured with a modified Boyden chamber assay. Specific inhibition of NEAT1 resulted in the decrease of the migratory and invasive ability of A549 cells and SK-MES-1 cells (Figures 2F,G). Knocking down of NEAT1 was further found to induce the downregulation of ki67, MMP2, and MMP9 expression level (Figures 2H,I).

### Knockdown of NEAT1 Inhibits Tumor Growth and Promote T Cell Infiltration in Xenograft Tumor Model

**FIGURE 3:**
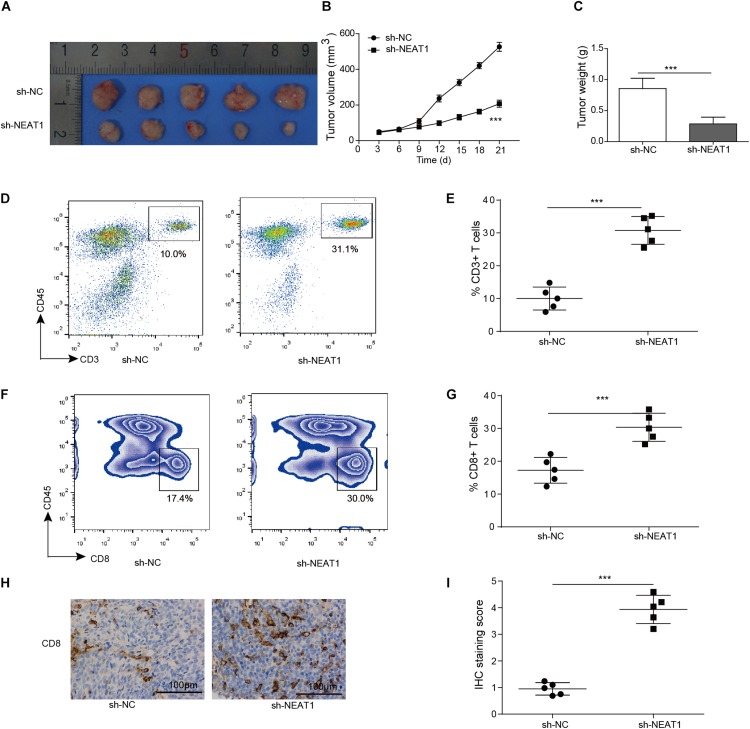
Inhibition of NEAT1 suppresses tumor growth *in vivo*. Panels **(A,B)** BALB/c mice were challenged with 10^6^ M109 cells subcutaneously, and tumor volume was assessed over time. **(C)** Tumor weight was recorded. **(D)** Representative plots of CD3 staining. Numbers indicated frequency of CD3^+^ T cells. **(E)** Frequency of tumor CD3^+^ T cells was analyzed by flow cytometry. **(F)** Representative plots of CD8 staining. Numbers indicate frequency of CD8^+^ T cells. **(G)** Frequency of tumor CD8^+^ T cells was analyzed by flow cytometry. **(H)** Representative image of CD8 staining by IHC. **(I)** IHC staining score of intratumor CD8^+^ T cells. ***p < 0.001. Data are expressed as the mean ± SD.

Given that silencing of NEAT1 could inhibit the cell viability of lung cancer cells *in vitro*, its function *in vivo* was also determined. Nuclear paraspeckle assembly transcript 1 knockdown M109 cells were subcutaneously inoculated into BALB/c mice for 21 days, and the sizes of tumors over time were determined. Knocking down of NEAT1 induced significant decrease in cell viability of M109 cells (Supplementary Figure S1). The data showed that the downregulation of NEAT1 showed antitumor effect in M109 syngeneic models (Figures 3A–C), suggesting that NEAT1 was able to promote lung cancer growth. To further confirm the antitumor immunity mediated by NEAT1 knockdown, the percentage of tumor-infiltrating cytotoxic T cells was next analyzed. The tumors with NEAT1 knockdown M109 cell harvested from mice had intratumoral elevation in CD45^+^ CD3^+^ T cells (Figures 3D,E) and CD4^–^ CD8^+^ T cells (Figures 3F,G) compared to tumor from control mice. Tumor-infiltrating CD8^+^ T cells were further examined using IHC staining. The IHC score showed that CD8^+^ T cells were upregulated when NEAT1 was inhibited (Figures 3H,I). Taken together, these data suggest NEAT1 knockdown drives an adaptive T cell tumor-specific immune response that results in tumor inhibition of lung cancer.

### Knockdown of NEAT1 Activates cGAS/STING Signaling and Induces Downstream Expression of CXCL10, CCL5, and IFNβ

**FIGURE 4:**
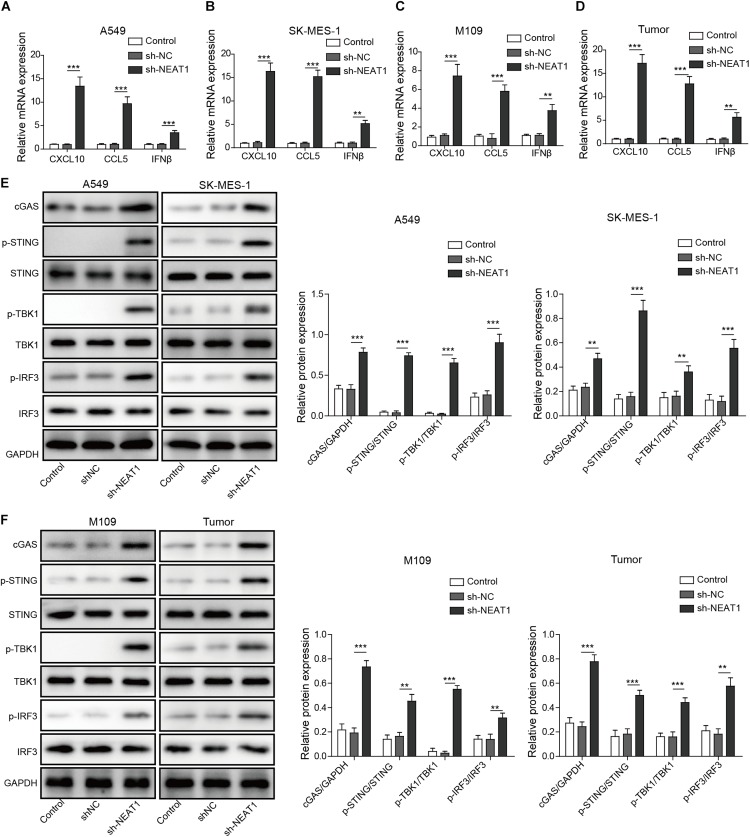
Inhibition of NEAT1 suppresses activate cGAS-STING signaling and inhibits CXCL10, CCL5, and IFNβ expression. **(A,B)** mRNA expression of CXCL10, CCL5, and IFNβ expression was detected in A549 cells and SK-MES-1 cells when transfected with the specific shRNA of NEAT1 by using RT-qPCR. **(C,D)** mRNA expression of CXCL10, CCL5, and IFNβ expression was detected by using RT-qPCR. **(E)** Protein level of cGAS-STING signaling-related molecules was detected in A549 cells and SK-MES-1 cells when silencing NEAT1 by using Western blot. The histogram indicated the quantification of the Western blot signals. **(F)** Protein level of cGAS-STING signaling-related molecules was detected in M109 cell lines and tumors when silencing NEAT1 by using Western blot. The histogram indicated the quantification of the Western blot signals. **p < 0.01, ***p < 0.001. Data are expressed as the mean ± SD.

Given that type I IFN signaling is necessary for the generation of CD8^+^ T cell responses against tumors (Fuertes et al., 2011), the chemokines and IFNβ mRNA and protein expression were examined in tumor cell lines. Enhanced mRNA and protein levels of CXCL10, CCL5, and IFNβ were observed following silencing of NEAT1 in human and mouse lung cancer cells as measured by qRT-PCR and Western blot analysis, respectively (Figures 4A–C and Supplementary Figure S2). Similarly, NEAT1 knockdown also resulted in upregulation of CXCL10, CCL5, and IFNβ expression *in vivo* (Figure 4D). Interferon β, CXCL10, and CCL5 are downstream cytokines regulated by cGAS-STING signaling (Ruangkiattikul et al., 2017). To investigate the effect of NEAT1 depletion on the cGAS-STING, the expression levels of cGAS-STING signaling-related proteins including TBK1 and IRF3 (Ruangkiattikul et al., 2017) were examined by Western blot assay. The results indicates that inhibition of NEAT1 results in the increase in cGAS, STING level, and upregulation of phosphorylation of TBK1 and IRF3 in both of cell lines and tumor samples (Figures 4E,F).

### NEAT1 Epigenetically Inhibits Expression of cGAS/STING and P53 via Binding to DNMT1

**FIGURE 5:**
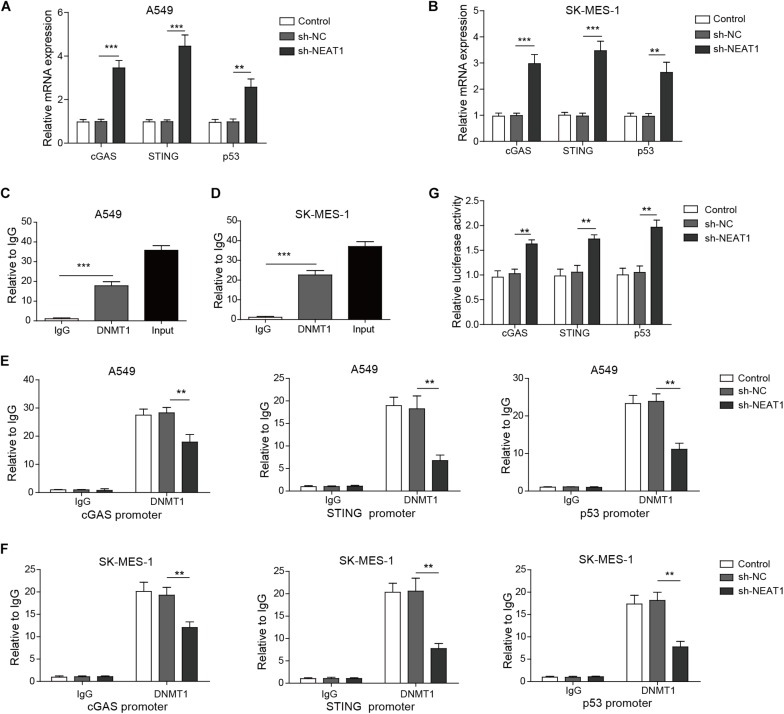
Nuclear paraspeckle assembly transcript 1 epigenetically suppresses P53, cGAS, and STING expression via binding to DNMT1. **(A,B)** mRNA expression of P53, cGAS, and STING was detected in A549 cells and SK-MES-1 cells when transfected with the NEAT1-specific shRNA, control sh-NC, or untransfected cells, by using RT-qPCR. **(C,D)** RIP assay confirmed that lncRNA NEAT1 could bind to DNMT1. **(E,F)** ChIP assay showed that DNMT1 could bind to the DNA in the promoter region of TP53, CGAS, and STING1 in A549 and SK-MES-1 cells, respectively. **(G)** Reporter activity of P53, CGAS, and STING1 after transfection with the specific shRNA for NEAT1 for 48 h. **p < 0.01, ***p < 0.001. Data are expressed as the mean ± SD.

Next, we investigated whether DNMT1 mediated the effect of NEAT1 on the expression of cGAS, STING, and P53 in lung cancer cells. The expression of cGAS, STING, and P53 was obviously upregulated in A549 cells and SK-MES-1 cell lines with lncRNA NEAT1 deficiency (Figures 5A,B). RNA immunoprecipitation assay confirmed that NEAT1 was able to bind to DNMT1 in both lung cancer cells (Figures 5C,D). The ChIP assay demonstrated that NEAT1 knockdown in A549 cells reduced the enrichment of DNMT1 on the TP53 promoter, CGAS promoter, and STING1 promoters (Figure 5E). Silencing of lncRNA NEAT1 in SK-MES-1 cells obtained the consistent results (Figure 5F). As shown in Figure 5G, the promoter activity of TP53, CGAS, and STING1 was upregulated during inhibition of DNMT1. Taken together, NEAT1 epigenetically inhibits expression of cGAS/STING and P53 via binding to DNMT1.

### NEAT1 Exhibited Oncogenic Effect at Least Partly Dependent on DNMT1

**FIGURE 6:**
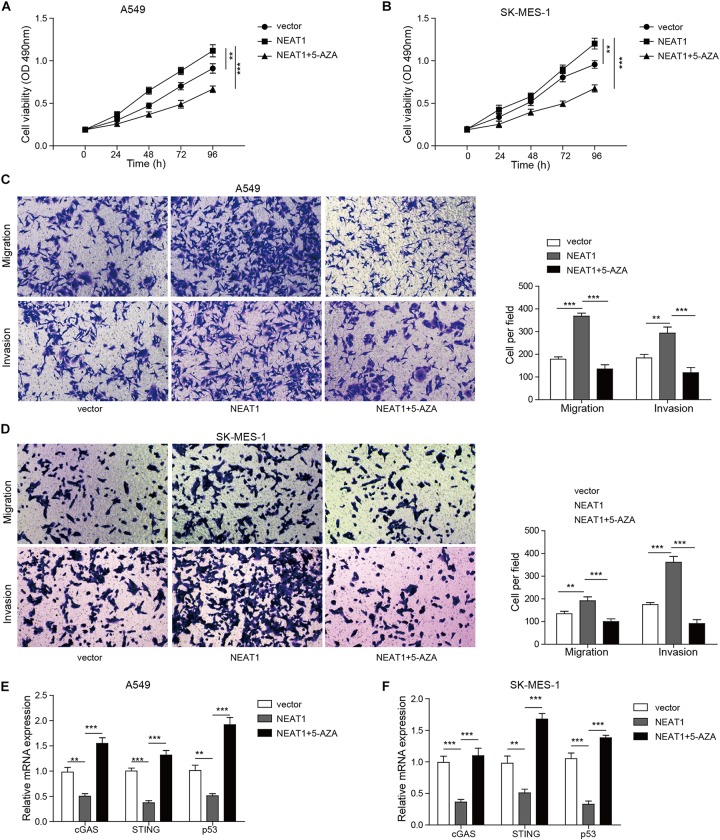
Nuclear paraspeckle assembly transcript 1 exhibited oncogenic effect at least partly dependent on DNMT1. **(A,B)** The proliferation of A549 cells and SK-MES-1 cells was analyzed after transfection with the specific shRNA for NEAT1 in the presence of DNMT1 inhibitor or not for 24, 48, 72, and 96 h by using MTT, respectively. **(C,D)** The cell migration and invasion of A549 cells and SK-MES-1 cells were analyzed after transfection with the specific shRNA for NEAT1 in the presence of DNMT1 inhibitor or not, respectively. **(E,F)** mRNA expression of P53, cGAS, and STING was detected in A549 cells and SK-MES-1 cells after transfection with the specific shRNA for NEAT1 in the presence of DNMT1 inhibitor or not, respectively, by using RT-qPCR. **p < 0.01, ***p < 0.001. Data are expressed as the mean ± SD.

Having demonstrated that NEAT1 promoted growth and migration/invasion of lung cancer cells, we speculated whether blocking of DNMT1 was able to reverse the protumor effect of lncRNA NEAT1. As shown in Figures 6A,B, overexpression of lncRNA NEAT1 enhanced viability of cancer cells in a time-dependent manner, whereas treatment with 5-aza, an inhibitor of DNMT1, abolished the effect of NEAT1 on cell survival. 5-Aza treatment also reversed the enhancement of cell migration/invasion by lncRNA NEAT1 (Figures 6C,D). Additionally, qPCR assay showed 5-aza treatment eliminated the inhibitory effect of lncRNA NEAT1 on the expression of P53, STING, and cGAS (Figures 6E,F).

## Discussion

In this report, we provided the evidence that NEAT1 promoted viability and migratory abilities of lung cancer cells by inhibiting P53. On the other hand, lncRNA NEAT1 exerted its inhibitory effect on cGAS-STING pathway, mediating the immune escape of tumor cells to T cells. Further studies revealed that NEAT1 exerted the above effects via binding with DNMT1.

Ectopic expression of NEAT1 has been reported in many types of cancer including breast cancer (Zhao et al., 2017), thyroid cancer (Li et al., 2017), cholangiocarcinoma (CCA) (Ju et al., 2018), hepatocellular carcinoma (Wang et al., 2017), ovarian cancer (Ding et al., 2017), colorectal cancer (Iguchi et al., 2015), pancreatic cancer (Huang et al., 2017), and osteosarcoma (OS) (Li and Cheng, 2018). The expression level of NEAT1 is associated with clinicopathological features of cancer patients (Yang et al., 2017). Nuclear paraspeckle assembly transcript 1 affects the proliferation, cell cycle progression, apoptosis, EMT, migration, and invasion of cancer cells (Dong et al., 2018). It is also involved in promotion of drug resistance. For example, LncRNA NEAT1 contributes to paclitaxel resistance of ovarian cancer cells by regulating ZEB1 expression via miR-194 (An et al., 2017). Consistently, we found that NEAT1 was upregulated in lung cancer tissues as well as lung cancer cell lines. Our results showed that NEAT1 was found to promote cell survival, migration, and invasion in lung cancer cells. Moreover, we further tested whether NEAT1 affected the specific markers for cell growth, migration, and invasion. The results showed that knockdown of NEAT1 attenuated the expression of proliferative marker Ki-67 and migrative markers MMP2/MMP9. The present study furthered the underlying mechanism of regulatory effect of NEAT1 on lung cancer.

The methylation of the tumor suppressor genes was modulated by DNA methyltransferase 1 (DNMT1) (Baylin and Jones, 2011). In lung cancer, DNA methylation may happen in the genome regardless of the methylation levels of the gene promoter region (Liu et al., 2016). LncRNAs were found to physically interact with DNMTs to modulate DNA methylation. Examples are *Dali*, which is important for neural differentiation and can regulate neural gene expression through binding with DNMT1 to modulate DNA methylation at distal target promoter (Zhao et al., 2016), and *Dum*, which regulates the differentiation program of skeletal myoblast via interacting with several DNMTs including DNMT1, DNMT3a, and DNMT3b (Wang et al., 2015). The interaction between NEAT1 and DNMT1 has been found in OS (Song et al., 2019). In the present study, we confirmed that NEAT1 regulated the cGAS-STING pathway, and P53 thus promotes cancer cell survival, migration, and invasion via binding to DNMT1.

Cyclic GMP-AMP synthase is an important cytosolic DNA sensor that plays a crucial role in triggering STING-dependent signal and inducing immune response. DNA released from bacteria activates cytosolic cGAS to produce cyclic cGAMP, which stimulated the STING-TBK-1-IRF3 signaling pathway to initiate transcription of downstream factors including IFNβ, CXCL10, and CCL5 (Ruangkiattikul et al., 2017). Upon stimuli by cytosolic DNA, cGAS-STING pathway triggered the expression of type I IFN in dendritic cells (DCs) or cancer cells (Bose, 2017). Type I IFN is a pleiotropic cytokine that is closely connected with cell senescence and inflammation response. Type I IFN showed multiple immune-stimulatory role in promoting the activation, migration, and maturation of several immune cells including DCs, T cells, and natural killer cells (Margolis et al., 2017). Furthermore, DNA from cancer cells can be transferred into DC cytoplasm and activate cGAS-STING-type I IFN pathway, which is critical to subsequent activation of T cells (Ng et al., 2018). In order to evade this DNA detection pathway to survive, there are several mechanisms utilized by tumor cells, yielding the defective cGAS-STING signaling in tumors, including decreased the protein level of STING and cGAS, hypermethylation of CGAS and STING1 promoter regions, and defective STING translocation to the Golgi where it normally signals (Khoo and Chen, 2018). In the present study, we for the first time found that cGAS-STING signaling was destructed by NEAT1. The expression of cGAS and STING was downregulated by NEAT1-mediated DNMT1 binding to the promoter region. Moreover, our *in vivo* study found that when NEAT1 was silenced, the tumor growth was retarded, and more importantly, cytotoxic T cells were elevated. These findings suggested that NEAT1 played a vital role in tumor immunity, which furthered the understanding of oncogenic mechanism of NEAT1 in lung cancer. In addition, we found that NEAT1 also mediated the inhibition of P53 by DNMT1. P53 is a major tumor suppressor in proliferation and migration. This result suggests that NEAT1 might regulate the progression of lung cancer by inhibiting P53 in addition to cGAS-STING signaling.

One of the reasons for limiting the implication of NEAT1 or lncRNA as target is that there is no suitable way to deliver drugs *in vivo*. Nanomedicine, the application of nanotechnology to medicine, enabled the development of nanoparticle therapeutic carriers (Baetke et al., 2015). Nanoparticles can be formulated to deliver drugs across several biological barriers. Tremendous progress has been made in recent years in delivering ncRNAs using nanoparticles (Javanmardi et al., 2018). The area needs further exploration to overcome challenges associated with *in vivo* delivery of ncRNAs with special emphasis of site-specific delivery, cellular uptake, and stability.

Collectively, NEAT1 was upregulated in lung cancer tissues and cell lines. The aberrant expression was closely associated with tumor stage and lymph node metastasis, suggesting that NEAT1 could be involved in the progression of lung cancer. However, we should admit that there are still obstacles for NEAT1 to be applied in diagnose or treatment of lung cancer due to the limitation of detection of non-coding RNAs. *In vitro* studies displayed that inhibition of NEAT1 with shRNA resulted in suppression of survival and migration/invasion of lung cancer cells mediated by targeting DNMT1/P53 signaling. On the other hand, NEAT1 was found to inhibit cGAS-STING signaling to help cells evade T cell tumor immunity.

## Data Availability Statement

The datasets generated for this study are available on request to the corresponding author.

## Ethics Statement

The studies involving human participants were reviewed and approved by the Ethical Committee of Central South University. The patients/participants provided their written informed consent to participate in this study. The animal study was reviewed and approved by the Institutional Animal Care and Use Committee of Central South University.

## Author Contributions

X-LL: guarantor of integrity of the entire study, study design, and manuscript review. FM: study concepts, definition of intellectual content, and manuscript editing. Y-YL: literature research and experimental studies. M-GD: data acquisition. L-HL: data analysis and statistical analysis. Y-CX: manuscript preparation.

## Conflict of Interest

The authors declare that the research was conducted in the absence of any commercial or financial relationships that could be construed as a potential conflict of interest.

## Supplementary Material

FIGURE S1 Down-regulation of NEAT1 suppresses the proliferation, migration and invasion of M109 mouse lung cancer cells. **(A)** mRNA expression of NEAT1 was detected in M109 cells when transfected with several specific shRNA of NEAT1 by using RT-qPCR. **(B)** The proliferation of M109 cells was analyzed after transfection with the specific shRNA for NEAT1 for 24, 48, 72, and 96 h by using MTT, respectively. **(C)** The cell migration and invasion of M109 cells was analyzed after transfection with the specific shRNA for NEAT1, respectively. ^∗^*p* ¡ 0.05; ^∗∗^*p* < 0.01; ^∗∗∗^*p* < 0.001. Data are expressed as the mean ± SD.

FIGURE S2 Inhibition of NEAT1 suppresses inhibit CXCL10, CCL5, and IFN expression. Production of CXCL10, CCL5, and IFN β expression was detected in **(A)** A549 cells, **(B)** SK-MES-1 cells and **(C)** M109 cells when transfected with the specific shRNA of NEAT1 by using ELISA ^∗∗^*p* < 0.01; ^∗∗∗^*p* < 0.001. Data are expressed as the mean ± SD.

FIGURE S3 Expression of NEAT1 with different status of TNM stage, lymph node metastasis, and smoke in TCGA database. **(A)** Expression of NEAT1 in different status of TNM stage in TCGA LUAD database. **(B)** Expression of NEAT1 in different status of lymph node metastasis in TCGA LUAD database. **(C)** Expression of NEAT1 in different status of smoke in TCGA LUAD database. **(D)** Expression of NEAT1 in different status of TNM stage in TCGA LUSC database. **(E)** Expression of NEAT1 in different status of lymph node metastasis in TCGA LUSC database. **(F)** Expression of NEAT1 in different status of smoke in TCGA LUSC database; ^∗^*p* < 0.05; “ns” denotes no significance.
