# Supplementary figures and images for: LncRNA NEAT1 Interacted With DNMT1 to Regulate Malignant Phenotype of Cancer Cell and Cytotoxic T Cell Infiltration via Epigenetic Inhibition of p53, cGAS, and STING in Lung Cancer
Source: Front Genet. 2020 Mar 31;11:250. doi: 10.3389/fgene.2020.00250 (PMC7136539; doi:10.3389/fgene.2020.00250)

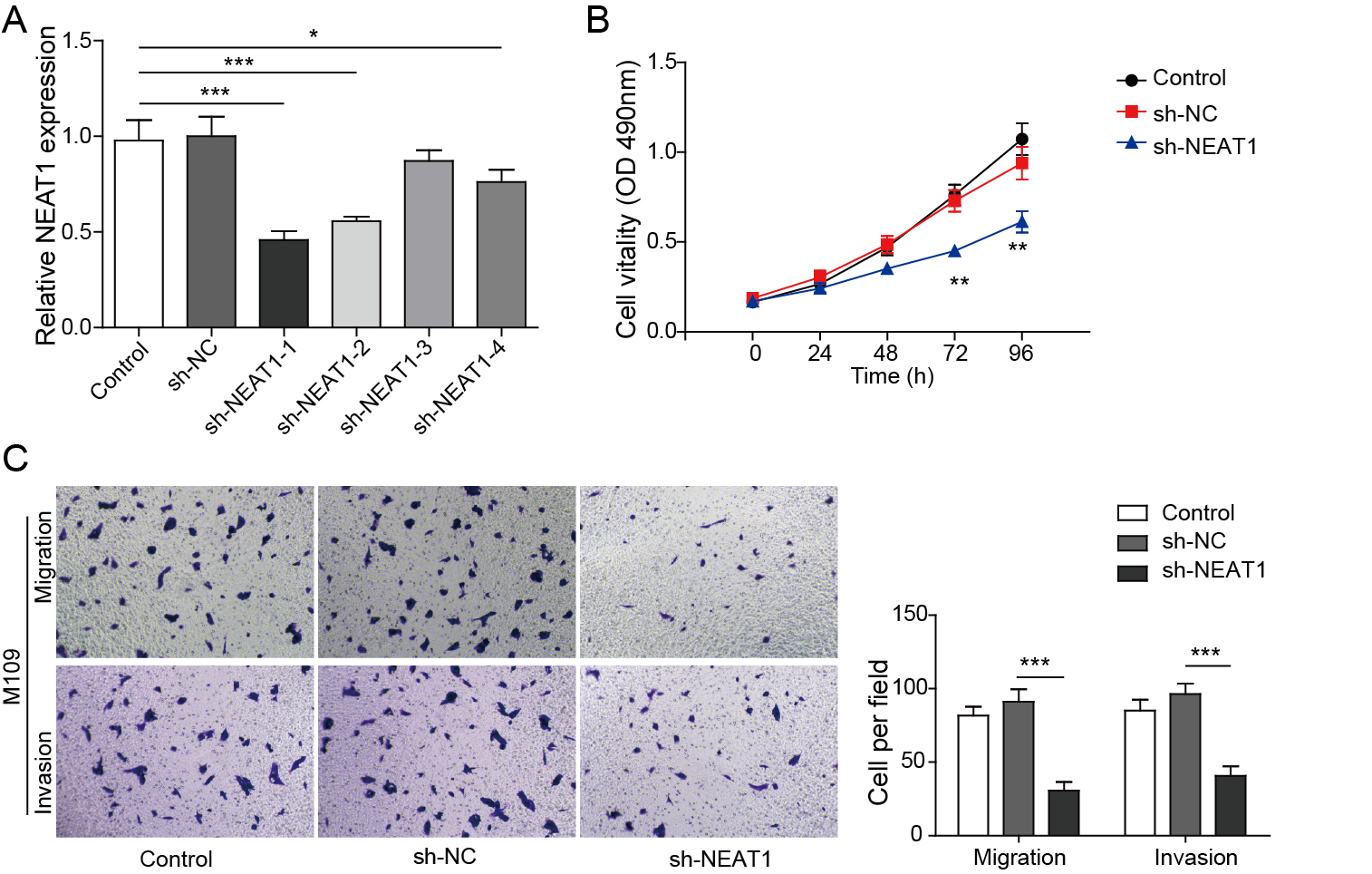

Supplement: FIGURE S1 — Down-regulation of NEAT1 suppresses the proliferation, migration and invasion of M109 mouse lung cancer cells. (A) mRNA expression of NEAT1 was detected in M109 cells when transfected with several specific shRNA of NEAT1 by using RT-qPCR. (B) The proliferation of M109 cells was analyzed after transfection with the specific shRNA for NEAT1 for 24, 48, 72, and 96 h by using MTT, respectively. (C) The cell migration and invasion of M109 cells was analyzed after transfection with the specific shRNA for NEAT1, respectively. ∗p ¡ 0.05; ∗∗p < 0.01; ∗∗∗p < 0.001. Data are expressed as the mean ± SD. [file Image_1.TIF]

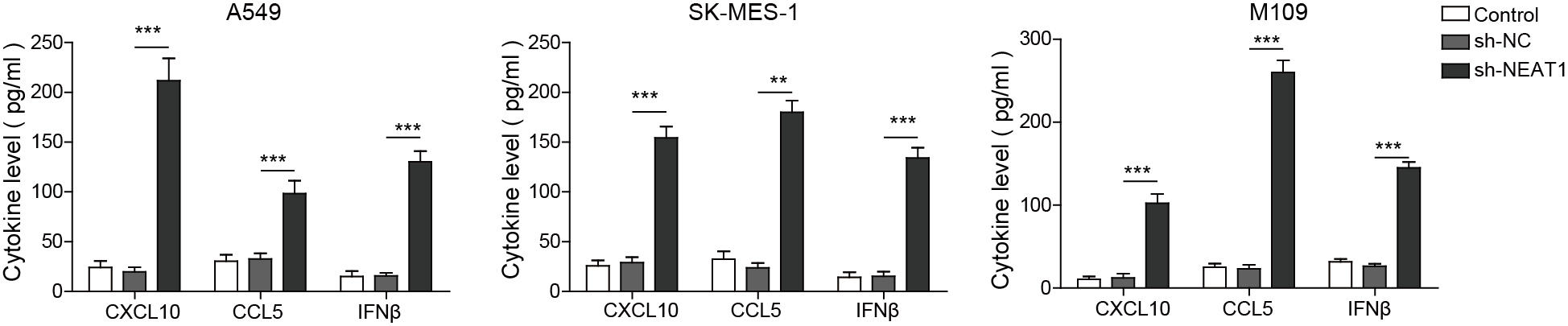

Supplement: FIGURE S2 — Inhibition of NEAT1 suppresses inhibit CXCL10, CCL5, and IFN expression. Production of CXCL10, CCL5, and IFN β expression was detected in (A) A549 cells, (B) SK-MES-1 cells and (C) M109 cells when transfected with the specific shRNA of NEAT1 by using ELISA ∗∗p < 0.01; ∗∗∗p < 0.001. Data are expressed as the mean ± SD. [file Image_2.TIF]

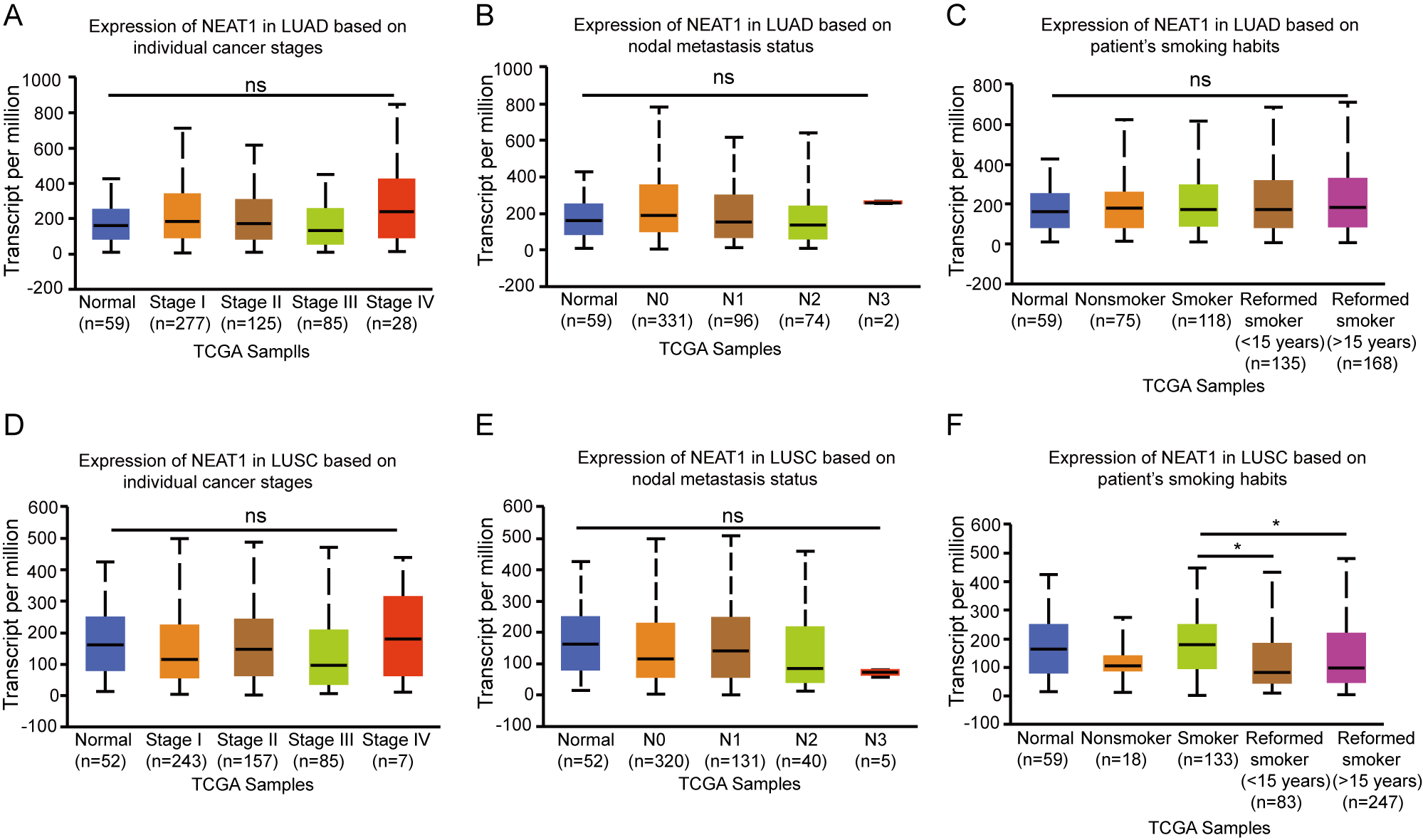

Supplement: FIGURE S3 — Expression of NEAT1 with different status of TNM stage, lymph node metastasis, and smoke in TCGA database. (A) Expression of NEAT1 in different status of TNM stage in TCGA LUAD database. (B) Expression of NEAT1 in different status of lymph node metastasis in TCGA LUAD database. (C) Expression of NEAT1 in different status of smoke in TCGA LUAD database. (D) Expression of NEAT1 in different status of TNM stage in TCGA LUSC database. (E) Expression of NEAT1 in different status of lymph node metastasis in TCGA LUSC database. (F) Expression of NEAT1 in different status of smoke in TCGA LUSC database; ∗p < 0.05; “ns” denotes no significance. [file Image_3.TIF]
